# Analysis of Phyllosphere Microorganisms and Potential Pathogens of Tobacco Leaves

**DOI:** 10.3389/fmicb.2022.843389

**Published:** 2022-04-29

**Authors:** Li-Gang Xiang, Han-Cheng Wang, Feng Wang, Liu-Ti Cai, Wen-Hong Li, Tom Hsiang, Zhi-He Yu

**Affiliations:** ^1^College of Agriculture, Yangtze University, Jingzhou, China; ^2^Guizhou Provincial Academician Workstation of Microbiology and Health, Guizhou Academy of Tobacco Science, Guiyang, China; ^3^Guizhou Institute of Plant Protection, Guizhou Academy of Agricultural Sciences, Guiyang, China; ^4^School of Environmental Sciences, University of Guelph, Guelph, ON, Canada; ^5^College of Life Sciences, Yangtze University, Jingzhou, China

**Keywords:** tobacco, microbial diversity, pathogen, metagenetic analysis, culture-dependent method

## Abstract

In the tobacco phyllosphere, some of the microbes may have detrimental effects on plant health, while many may be neutral or even beneficial. Some cannot be cultivated, so culture-independent methods are needed to explore microbial diversity. In this study, both metagenetic analysis and traditional culture-dependent methods were used on asymptomatic healthy leaves and symptomatic diseased leaves of tobacco plants. In the culture-independent analysis, asymptomatic leaves had higher microbial diversity and richness than symptomatic leaves. Both asymptomatic and symptomatic leaves contained several potentially pathogenic bacterial and fungal genera. The putative bacterial pathogens, such as species of *Pseudomonas*, *Pantoea*, or *Ralstonia*, and putative fungal pathogens, such as species of *Phoma*, *Cladosporium*, *Alternaria*, *Fusarium*, *Corynespora*, and *Epicoccum*, had a higher relative abundance in symptomatic leaves than asymptomatic leaves. FUNGuild analysis indicated that the foliar fungal community also included endophytes, saprotrophs, epiphytes, parasites, and endosymbionts. PICRUSt analysis showed that the dominant functions of the bacterial community in a symptomatic leaf were cellular processes and environmental information processing. In the other five foliar samples, the dominant functions of the bacterial community were genetic information processing, metabolism, and organismal systems. In the traditional culture-dependent method, 47 fungal strains were isolated from 60 symptomatic tobacco leaf fragments bearing leaf spots. Among them, 21 strains of *Colletotrichum* (29%), *Xylariaceae* (14%), *Corynespora* (14%), *Pestalotiopsis* (10%), *Alternaria* (10%), *Epicoccum* (10%), *Byssosphaeria* (5%), *Phoma* (5%), and *Diaporthe* (5%) all fulfilled Koch’s postulates and were found to cause disease on detached tobacco leaves in artificial inoculation tests. Symptoms on detached leaves caused by three strains of *Corynespora cassiicola* in artificial inoculation tests were similar to the original disease symptoms in the tobacco field. This study showed that the combined application of culture-dependent and independent methods could give comprehensive insights into microbial composition that each method alone did not reveal.

## Introduction

Tobacco (*Nicotiana tabacum* L.) is an annual, leafy, solanaceous plant, and one of the most widely cultivated non-food crops in the world (Chen et al., 2020). In China, the annual planting area of tobacco is more than 1 million hectares, and the annual output is more than 2 million tons (Yan et al., 2021). Tobacco leaf diseases are one of the main challenges in tobacco cultivation, and these can be divided into physiological, bacterial, fungal, and viral diseases. A typical physiological leaf disease of tobacco is weather fleck caused by high levels of ozone or air pollution (Macdowall et al., 1964). The fungal leaf diseases of tobacco include brown spot (Stavely and Slana, 1975), and powdery mildew (Cai et al., 2017), among others. Important bacterial leaf diseases of tobacco include angular leaf spot disease (Fromme and Murray, 1919) and wildfire disease (Cai et al., 2019). Important viral leaf diseases of tobacco are caused by tobacco mosaic virus (Scholthof, 2004), etch virus (Hari, 1981), and cucumber mosaic virus (Wagih et al., 2021).

The phyllosphere is colonized by specific microorganisms as an essential plant-associated habitat (Vorholt, 2012). However, the environmental conditions of the phyllosphere have great impact on foliar microbial populations. Phyllosphere microorganisms face harsh environmental conditions including nutrient and free water limitations and high ultraviolet radiation (Vorholt, 2012). Even with such poor growth conditions, there are numerous microorganisms that can colonize and exploit the phyllosphere, including bacteria, fungi, yeast, protists, algae, and bacteriophages. According to Vorholt (2012), about 10^6^–10^7^ cells reside on 1 cm^2^ of leaf surface. Within these communities, some microorganisms may be beneficial or neutral to the host plant, while others may be pathogenic and antagonistic to the host plant. Pathogens can invade plant leaves through wounds, stomates, hydathodes or other openings (Hugouvieux et al., 1998).

In the past few decades, plant pathogens have traditionally been discovered and identified based on culture-dependent methods. Because these are selective methods, the results can be influenced by many factors, including surface sterilization approaches, culture medium, and incubation conditions (Dissanayake et al., 2018). Therefore, the results obtained by traditional culture-dependent method cannot comprehensively reflect the numbers and functions of foliar microorganisms or pathogens. Metagenetic analysis involving high-throughput sequencing as a culture-independent method has also been used to investigate phyllosphere microbial community and diversity (Luo et al., 2019). However, species resolution of this technique is strongly influenced by databases available since it will not identify species that are not represented in the database. Furthermore, because of variability within species and similarity between species especially of the same genus, the taxonomic assignments at the commonly used threshold of 97% similarity are not entirely accurate for species level identification (Nilsson et al., 2008). In other words, the taxonomic results derived from metagenetic analysis may be reasonable only to resolve to the genus level (Dissanayake et al., 2018). Species-level classification may be obtained using species-specific primers or by analyzing specific loci, but these methods are labor-intensive and complex.

The aims of this study included (i) characterizing fungi and bacteria associated with asymptomatic healthy leaves and symptomatic diseased leaves of tobacco plants using the internal transcribed spacer (ITS) region and the partial 16S rRNA gene sequence, (ii) isolating potential fungal pathogens from symptomatic leaves of an unknown leaf spot disease of tobacco, and (iii) comparing the results for fungi from metagenetic analysis vs. culturing and ITS sequencing from the same samples.

## Materials and Methods

### Sampling Strategy and Sites

**FIGURE 1:**
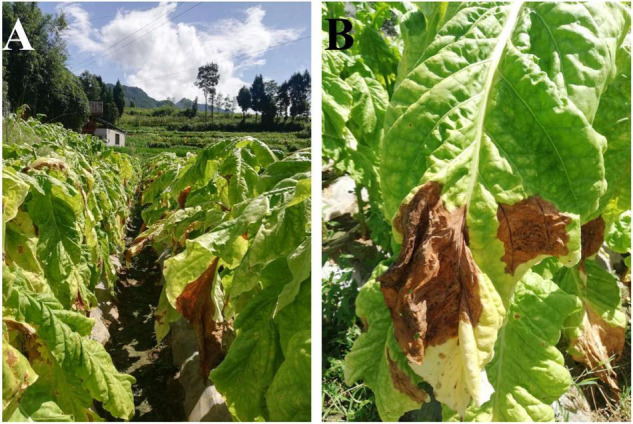
Field symptoms of diseased tobacco plants **(A)** and leaves **(B)**.

Samples were collected from Tongren City (27.94N, 108.26E), Guizhou province, China in July 2020. In a field where plants of tobacco cultivar Yunyan K326 were mature, six plants at least 3 m apart were randomly chosen for sampling. Three of these plants had leaves with large yellow-brown irregular spots and lesions covering more than 25% of each leaf (Figure 1), and a leaf from each was randomly selected as representative samples for symptomatic leaves (GB1–GB3). The other three tobacco plants were healthy and used to collect asymptomatic leaves (JK1–JK3). Each leaf was divided into two sets of samples, one for fungal isolation (stored at 4°C) and another for microbial community structure and diversity analysis (stored at −80°C).

### Culture-Independent Method

#### DNA Extraction, Polymerase Chain Reaction Amplification and Metagenetic Analysis

Leaf samples were not disinfected so as to obtain both endophytes and epiphytes. To ensure homogeneous sampling, the leaf tissue was cut into pieces and then 0.5 g of tissue was randomly taken for DNA extraction (only necrotic leaf tissues were collected as symptomatic samples). Total genomic DNA was extracted using a FastDNA^®^ Spin Kit (MP Biomedicals, Santa Ana, CA, United States) based on the manufacturer’s protocol. DNA quantity and quality were assessed using agarose gel electrophoresis, and the concentrations estimated using the NanoDrop-2000 (Thermo Fisher Scientific, Waltham, MA, United States). DNA was diluted to 30 ng/μL using sterile water. After DNA extraction, the bacterial V4 region of the 16S rRNA gene was amplified using primers 515F (5′-GTGYCAGCMGCCGCGGTAA-3′) and 806R (5′-GGACTACHVGGGTWTCTAAT-3′) (Caporaso et al., 2011), and the fungal ITS1 region was amplified using primers ITS1 (5′-TCCGTAGGTGAACCTGCGG-3′) and ITS2 (5′-GCTGCGTTCTTCATCGATGC-3′) (Zhang et al., 2010). The polymerase chain reaction (PCR) programs and total reaction volumes for ITS and 16S rRNA regions followed Wei et al. (2018). The PCR products were detected with 2% agarose gel electrophoresis using the DL2000 marker (TaKaRa, Dalian, China) for quantity examination, and purified with the Qiagen Gel Extraction Kit (Qiagen, Düsseldorf, Germany). Sequencing libraries were generated using the TruSeq^®^ DNA PCR-Free Sample Preparation Kit (Illumina, CA, United States) following manufacturer’s recommendations, and index codes were added. The library quality was assessed on the Qubit@ 2.0 Fluorometer (Thermo Fisher Scientific, Waltham, MA, United States) and Agilent 2100 Bioanalyzer (Agilent Technologies, Palo Alto, CA, United States). Finally, the library was sequenced on an Ion S5 XL platform (Thermo Fisher Scientific, Waltham, MA, United States) at Novogene Bioinformatics Technology Co., Tianjin, China, targeting 250 bp paired-end reads.

#### Data Processing and Statistical Analyses

Paired-end reads were merged using FLASH V1.2.7 (overlap ≥10 bp, maximum mismatch density of 0.25) (Magoč and Salzberg, 2011). Quality filtering of raw reads was performed with specific criteria to obtain clean reads (Bokulich et al., 2013) based on the QIIME V1.9.1 quality control process (*q* ≤ 19) (Caporaso et al., 2010). The UCHIME algorithm (Edgar et al., 2011) was used to detect chimeric sequences, which were removed (Haas et al., 2011). After these operations, the sequences were considered clean reads. ITS reads were compared against the UNITE database (ver. 12.01.2017), and 16S rRNA reads were compared against the Silva database (ver. 132).

Sequences with ≥97% similarity were assigned to the same operational taxonomic unit (OTU) using Uparse V7.0.1001 software (Edgar, 2013). Subsequently, for each representative sequence of each OTU, the UNITE and Silva databases were used based on the Mothur algorithm to annotate taxonomic information. Duncan’s multiple range tests were performed in SPSS V23.0 (IBM, Armonk, NY, United States) at *p* < 0.05 to compare relative abundance between microbial genera of symptomatic versus asymptomatic leaves. The OTU tables of fungal and bacterial communities were normalized by rarefying to 8000 sequences per sample for alpha and beta diversity analyses. Finally, the estimates of alpha and beta diversity of microbial communities were calculated using QIIME V1.7.0 and V1.9.1, respectively, and displayed with R software V2.15.3. The alpha index comparison between symptomatic versus asymptomatic leaves used the Wilcoxon signed rank test at *p* < 0.1. Principal co-ordinates analysis (PCoA) of unweighted UniFrac distance were calculated based on Bray-Curtis dissimilarity matrix. The FUNGuild database^1^ was used to analyze fungal trophic mode and guild (Nguyen et al., 2016). PICRUSt^2^ was used to predict bacterial functionality based on the Greengene database (Langille et al., 2013). The relationship of the different genera was demonstrated using network analysis based on Spearman’s rank analysis. The co-occurrence patterns of tobacco phyllosphere microbial genera were assessed based on strong (*r* > 0.6) and significant correlations (*p* < 0.05) (Li and Wu, 2018).

### Culture-Dependent Method

#### Fungal Isolation

Each symptomatic leaf was cut into 20 small pieces (5 mm × 5 mm). All tissue pieces were surface disinfected for 30 s in 75% ethanol, and then sterilized with 1% sodium hypochlorite (NaOCl) for 1 min and washed three times in sterile distilled water (Li et al., 2017). Finally, the tissues were placed onto potato dextrose agar (PDA) and cultured at 25°C. Plates were checked daily for visible hyphal growth up to 1 week, and once the hyphal tips were visible around the leaf tissue, some tips were transferred to fresh PDA for purification via serial hyphal tip transfer. All purified isolates were stored on PDA slants at 4°C.

#### Molecular Identification of Isolated Strains

Mycelia cultured on PDA for a week were collected, and their genomic DNA was extracted using the DNeasy^®^ PowerLyzer^®^ Microbial Kit (Qiagen, Düsseldorf, Germany). The DNA concentration was assessed using the NanoDrop-2000, and diluted to 30 ng/μL using sterile water. The ITS was amplified with primers ITS1 (5′-TCCGTAGGTGAACCTGCGG-3′) and ITS4 (5′-TCCTCCGCTTATTGATATGC-3′) (White et al., 1990). The total reaction volume was 25.0 μL, including 10.5 μL of Taq PCR Master Mix, 1.0 μL each of primer (10 μM), 1.0 μL of template DNA, and 11.5 μL of double distilled water. The PCR program was used as followed: Denaturation at 94°C for 3 min, denaturation at 94°C for 30 s, annealing at 56°C for 30 s, extension at 72°C for 45 s for 30 cycles, and final extension at 72°C for 5 min. The amplified fragments (500–750 bp) were sequenced in both directions by Sango Biotech Co., Ltd., Shanghai, China and consensus sequences were derived and edited with DNAMAN (version 5.2.2). The sequences were compared to the NCBI nr database using BLASTn and the top matching hits showing >98% identity were chosen to represent the species.

#### Pathogenicity Testing

Fresh young detached tobacco leaves without visible disease symptoms were obtained. They were surface disinfected with 75% ethanol for 30 s, 1% NaOCl for 1 min, and finally washed three times using distilled water. A sterile piece of cotton was wrapped around the petiole of each detached leaf and moistened to prevent wilt. These leaves were each gently wounded with a sterile needle in a 4 mm^2^ area by lightly scratching, and a 5-mm-diameter mycelial plug was placed on the wound. Each treatment (isolated strain) was tested on three detached leaves, and fresh PDA plugs without hyphae were used as non-inoculated control. After inoculation, detached leaves were placed in plastic boxes and incubated at 28°C for 10 days in the dark, and then assessed for disease in terms of lesion area and color. Pathogenicity testing was repeated twice.

## Results

### Sequence Data

A total of 263,626 and 243,453 clean bacterial 250-bp reads were obtained from the asymptomatic and symptomatic samples, respectively. The paired-end reads were merged and then clustered into 304 and 272 OTUs using a cut off of 97% similarity. The OTUs were compared against the Silva database for identity. For fungi, there were 238,329 clean reads from asymptomatic samples, and paired-end reads were merged and clustered into 77 OTUs. The 269,671 clean reads from symptomatic samples were processed and clustered into 149 OTUs (Table 1). The fungal OTUs were compared against the UNITE database. The fungal and bacterial raw sequences of each sample have been deposited in the SRA database under project number PRJNA722239.

**TABLE 1:** Metagenetic sequence data from different samples of tobacco leaves.

Kingdom	Sample name^1^	Raw reads	Clean reads	Bases (nt)	Average length (nt)	Number of OTUs
Bacteria	JK1	96,717	91,435	26,484,462	406	98
Bacteria	JK2	90,456	85,532	26,878,593	406	108
Bacteria	JK3	91,273	86,659	24,974,300	406	98
Bacteria	GB1	83,895	79,231	25,489,125	406	110
Bacteria	GB2	88,806	83,456	26,754,105	424	96
Bacteria	GB3	84,731	80,766	26,637,397	406	66
Fungi	JK1	62,893	61,822	18,501,997	300	29
Fungi	JK2	98,602	96,874	18,344,049	300	24
Fungi	JK3	81,344	79,633	20,840,030	299	24
Fungi	GB1	90,234	88,237	17,955,023	294	59
Fungi	GB2	85,798	85,388	13,777,273	204	41
Fungi	GB3	99,549	96,046	18,698,034	291	49

*^1^JK, asymptomatic leaf; GB, symptomatic leaf.*

### Microbial Community Composition

**FIGURE 2:**
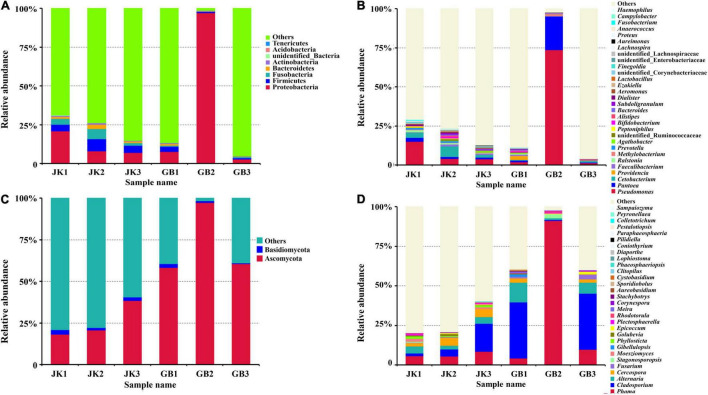
Microbial community composition of different samples (asymptomatic leaves JK1, JK2, JK3; symptomatic leaves GB1, GB2, GB3) at the phylum [**(A)** for bacteria, **(C)** for fungi] and genus [**(B)** for bacteria, **(D)** for fungi] levels. The relative abundance of phyla or genera making up less than 0.01% were classified as “Others.”

The influence of disease on microbial communities of tobacco leaves was assessed in this study. A rich microbial community was observed in both symptomatic and asymptomatic leaves. The “Other” group in Figure 2 contains all microorganisms that were not identified or whose relative abundance was less than 0.1%. Aside from “others,” the dominant identified bacterial phyla in both types of leaves were Proteobacteria, Firmicutes, Fusobacteria, Bacteroidetes, and Actinobacteria, with relative abundances ranging from 0.4 to 36% (Figure 2A). The major fungal phylum found was Ascomycota which for some of the samples exceeded “Others.” Basidiomycota were also present at a much lower levels in each sample. The relative abundance of Ascomycota in asymptomatic or symptomatic leaves was 26 ± 11% or 72 ± 22%, respectively; and for Basidiomycota, it was 2.3 ± 0.7% or 1.2 ± 1.0%, respectively (Figure 2C).

The relative abundance of the top 30 identified bacterial or fungal genera are shown in Figures 2B,D, respectively. Among the top 30 bacterial genera, most were non-pathogenic, and only some species from three of these genera have been classified as plant pathogens, namely *Pseudomonas*, *Pantoea*, and *Ralstonia*. The relative abundances of these three genera in symptomatic leaves were 25.67, 7.65, and 0.24%, respectively, and in asymptomatic leaves, they were 7.61, 1.60, and 0.73%, respectively. In addition to these genera, *Prevotella*, *Peptoniphilus*, *Ezakiella*, Corynebacteriaceae (family), *Proteus*, *Anaerococcus*, and *Campylobacter* were exclusively found in asymptomatic leaves, although in lower abundance (<0.7%).

Compared with bacterial genera, the top 30 identified fungal genera had more potential plant pathogens (Table 2). A total of 15 genera containing plant pathogens were detected in asymptomatic or symptomatic leaves. Among them, only the relative abundance of *Cercospora*, *Moesziomyces*, *Phyllosticta*, and *Plectosphaerella* in asymptomatic leaves was higher than their counterparts in symptomatic leaves. When counts were combined from asymptomatic or symptomatic leaves, the pathogenic genera with relative abundance over 1% included *Phoma* (6.6%/35.1%), *Cladosporium* (8.0%/23.9%), *Alternaria* (3.7%/6.9%), *Cercospora* (4.1%/1.5%), and *Fusarium* (0.22%/1.18%). Fungal genera which were found only on asymptomatic leaves included *Clitopilus* and *Phaeosphaeriopsis*, and those found only on symptomatic leaves included *Gibellulopsis*, *Meira*, *Stachybotrys*, *Aureobasidium*, and *Cystobasidium*.

**TABLE 2:** The top 30 dominant taxa and their relative abundance (%) of fungal and bacterial community OTUs based on metagenetic analysis.

Bacteria			Fungi		

Taxonomy	JK	GB	Taxonomy	JK	GB
*Pseudomonas*	7.61	25.67	*Phoma*	6.56	35.05
*Pantoea*	1.60	7.65	*Cladosporium*	7.96	23.87
*Cetobacterium*	3.95	0.43	*Alternaria*	3.66	6.88
*Providencia*	0.00	0.83	*Cercospora*	4.09	1.51
*Faecalibacterium*	0.82	0.41	*Fusarium*	0.22	1.18
*Ralstonia*	0.73	0.24	*Stagonosporopsis*	0.22	0.86
*Methylobacterium*	0.35	0.45	*Moesziomyces*	0.97	0.22
*Prevotella*	0.68	–	*Gibellulopsis*	–	0.54
*Agathobacter*	0.46	0.12	*Phyllosticta* *	1.08	0.11
*Ruminococcaceae*	0.46	0.30	*Golubevia*	0.75	0.22
*Peptoniphilus*	0.48	–	*Epicoccum*	0.32	0.54
*Bifidobacterium*	0.39	0.26	*Plectosphaerella*	0.54	0.43
*Alistipes*	0.31	0.25	*Rhodotorula*	0.32	0.32
*Bacteroides*	0.51	0.31	*Meira*	–	0.22
*Subdoligranulum*	0.42	0.21	*Corynespora*	0.22	0.32
*Dialister*	0.42	0.04	*Stachybotrys*	–	0.11
*Aeromonas*	0.33	0.05	*Aureobasidium*	–	0.11
*Ezakiella*	0.37	–	*Sporidiobolus*	0.11	0.11
*Lactobacillus*	0.15	0.04	*Cystobasidium*	–	0.11
*Corynebacteriaceae*	0.19	–	*Clitopilus*	0.11	–
*Finegoldia* *	0.28	0.00	*Phaeosphaeriopsis*	0.11	–
*Enterobacteriaceae*	0.18	0.03	*Lophiostoma*	<0.01	<0.01
*Lachnospiraceae*	0.21	0.12	*Diaporthe*	<0.01	<0.01
*Lachnospira*	0.19	0.07	*Coniothyrium*	<0.01	<0.01
*Aureimonas*	0.13	0.15	*Pilidiella*	<0.01	<0.01
*Proteus*	0.11	–	*Paraphaeosphaeria*	<0.01	<0.01
*Anaerococcus*	0.13	–	*Pestalotiopsis*	<0.01	<0.01
*Fusobacterium*	0.14	0.02	*Colletotrichum*	<0.01	<0.01
*Campylobacter*	0.11	–	*Peyronellaea*	<0.01	<0.01
*Haemophilus*	0.07	0.02	*Sampaiozyma*	<0.01	<0.01

*Significantly different at p = 0.05 based on three replicates for each mean. “–,” “the genus was not detected in the leaves.” JK, asymptomatic leaf; GB, symptomatic leaf.

### Microbial Community Diversity

Estimates of indices of richness and diversity for fungi and bacteria in asymptomatic and symptomatic leaves are shown in Table 3. For bacterial community analysis, the asymptomatic leaves show trends of higher Shannon–Weaver index, Simpson index, Chao1 index, and ACE index compared to the symptomatic leaves, but there were no statistically significant differences. For fungal community analysis, the asymptomatic leaves showed trends of higher Shannon–Weaver index and Simpson index, and a lower Chao1 index and ACE index compared to the symptomatic leaves, but there were no statistically significant differences.

**TABLE 3:** Alpha diversity indexes of phyllosphere microbial community.

Kingdom	Sample name^1^	Shannon	Simpson	Chao1	ACE	Goods_coverage
Bacteria	JK1	2.011	0.504	95.5	101.2	0.998
Bacteria	JK2	2.089	0.450	106.4	108.3	0.999
Bacteria	JK3	1.327	0.275	93.7	94.5	0.998
Bacteria	GB1	1.225	0.249	124.2	121.5	0.996
Bacteria	GB2	1.218	0.41	97.1	99.3	0.997
Bacteria	GB3	0.477	0.094	69.2	76.1	0.998
Fungi	JK1	3.23	0.806	28.6	30.1	0.987
Fungi	JK2	3.131	0.826	25.5	27.4	0.981
Fungi	JK3	3.224	0.851	26.3	29.2	0.977
Fungi	GB1	2.815	0.762	60.5	82.5	0.939
Fungi	GB2	0.698	0.165	13.5	16.1	0.987
Fungi	GB3	3.054	0.795	35.1	43.5	0.965

*^1^JK, asymptomatic leaf; GB, symptomatic leaf.*

**FIGURE 3:**
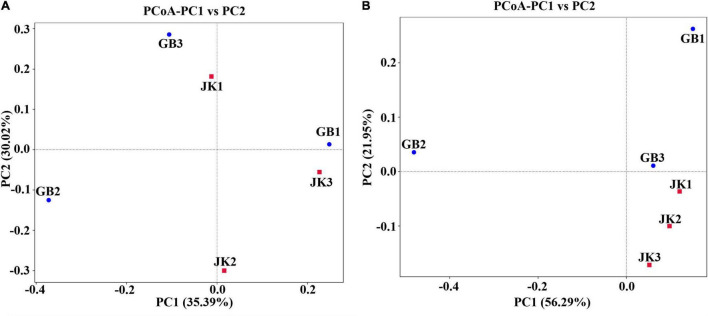
Principal Co-ordinate Analysis (PCoA) of the bacterial **(A)** and fungal **(B)** communities of different samples (asymptomatic leaves JK1, JK2, JK3; symptomatic leaves GB1, GB2, GB3).

Principal co-ordinates analysis for bacterial communities did not show separate clustering for symptomatic vs. asymptomatic samples (Figure 3A), which meant that the bacterial community composition was similar between symptomatic and asymptomatic groups. However, for fungal communities, the asymptomatic samples did cluster together in a PCoA quadrant, while the symptomatic samples were more scattered (Figure 3B), which implied that there were fewer differences in fungal community composition of asymptomatic leaves compared to symptomatic leaves.

### Microbial Community Functional Characteristics

**FIGURE 4:**
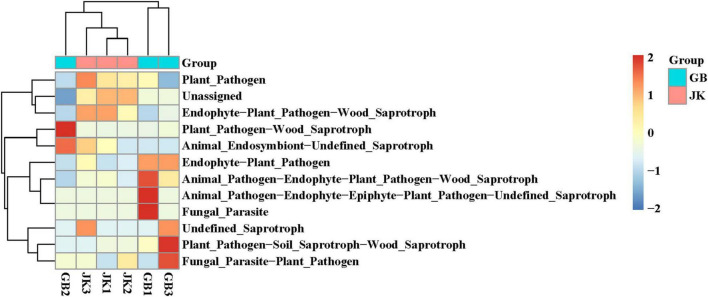
Dendrogram of fungal functional categories in symptomatic (GB1, GB2, GB3) and asymptomatic samples (JK1, JK2, JK3) analyzed using FUNGuild.

FUNGuild was used to predict the ecological functions of fungi in the samples based on their composition (Figure 4). The dominant ecological functions of fungal communities in asymptomatic leaves were classified as endophyte, plant pathogen, and wood saprotroph (JK1); fungal parasite and plant pathogen (JK2); and plant pathogen, endophyte, wood saprotroph, and animal endosymbiont (JK3). The dominant ecological functions of fungal communities in symptomatic leaves were fungal parasite, animal pathogen, endophyte, epiphyte, plant pathogen, and saprotroph (GB1); animal endosymbiont, saprotroph, and plant pathogen (GB2); and fungal parasite, plant pathogen, soil saprotroph, wood saprotroph, and endophyte (GB3).

**FIGURE 5:**
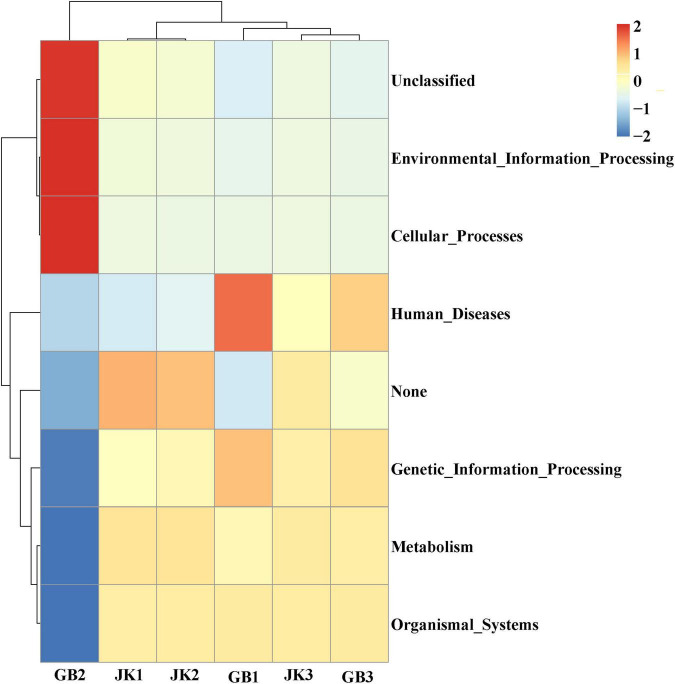
Dendrogram of bacterial functional categories in symptomatic (GB1, GB2, GB3) and asymptomatic samples (JK1, JK2, JK3) analyzed using PICRUSt.

PICRUSt was designed to estimate the gene families in metagenetic analysis from bacteria or archaea identified using 16S rRNA sequencing (Figure 5). In this study, genetic information processing, metabolism, and organismal systems were the common bacterial community functions in JK1, JK2, JK3, GB1, and GB3. In addition to the common functions, these were specific bacterial community in symptomatic leaves: unclassified, environment information processing, cellular processes, and human disease.

### Co-occurrence Network Analysis

**FIGURE 6:**
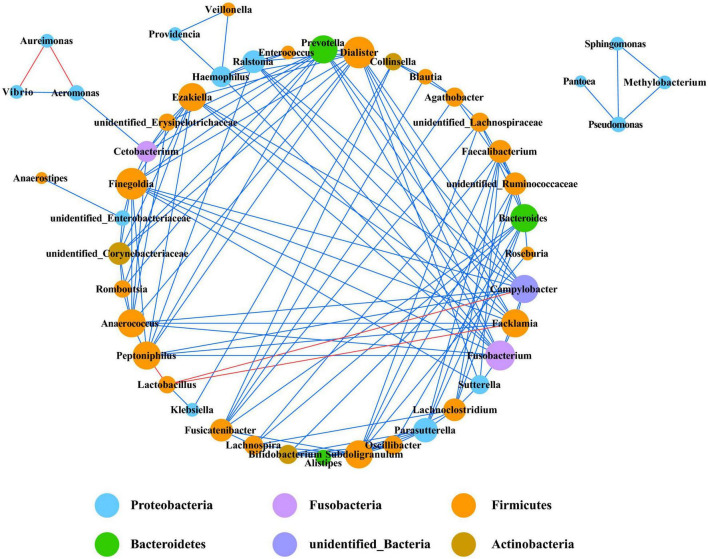
Co-occurrence network of bacteria at genera level. Only *p* values less than 0.05 and corrections coefficients higher than 0.6 were constructed in the network. The size of each node is proportional to the number of connections, the color of each node is correlates with taxonomy. Blue edges indicate positive correlations, and red edges indicate negative correlations.

Co-occurrence network analysis is widely used to study the interaction of organisms in complex microbial communities. A total of five strong negative correlations and 124 strong positive correlations were identified from 46 bacterial genera (Figure 6). The co-occurring genera were distributed in Firmicutes (50%, targeted nodes/total nodes), Proteobacteria (30.4%), Actinobacteria (6.5%), Bacteroidetes (6.5%), Fusobacteria (4.4%), and unidentified_Bacteria (2.2%). *Dialister*, *Peptoniphilus*, and *Prevotella* were identified as the top three genera based on high centrality score. The only five strong negative correlations in the bacterial communities were *Peptoniphilus*-*Lactobacillus*, *Aeromonas*-*Aureimonas*, *Lactobacillus*-*Campylobacter*, *Lactobacillus*-*Facklamia*, and *Aureimonas*-*Vibrio*.

**FIGURE 7:**
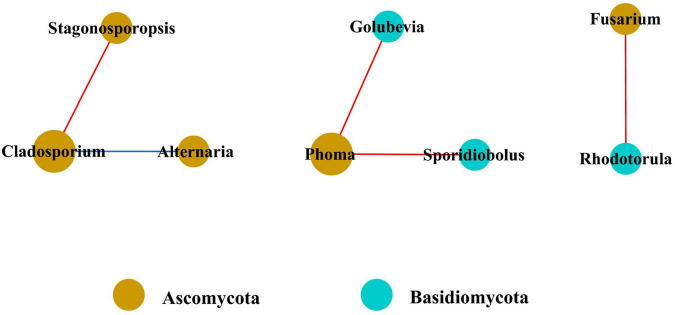
Co-occurrence network of fungi at genera level. Only *p* values less than 0.05 and corrections coefficients higher than 0.6 were constructed in the network. The size of each node is proportional to the number of connections, the color of each node is correlates with taxonomy. Blue edges indicate positive correlations, and red edges indicate negative correlations.

In the fungal community, only eight genera had strong and significant correlations (Figure 7) (*p* < 0.05, *r* > 0.6). There were significant negative correlations between *Cladosporium* and *Stagonosporopsis*, *Phoma* and *Golubevia*, *Phoma* and *Sporidiobolus*, and *Rhodotorula* and *Fusarium*. Only *Cladosporium* and *Alternaria* had a strong and significant positive correlation in co-occurrence network analysis.

### Culture-Based Fungal Diversity

**FIGURE 8:**
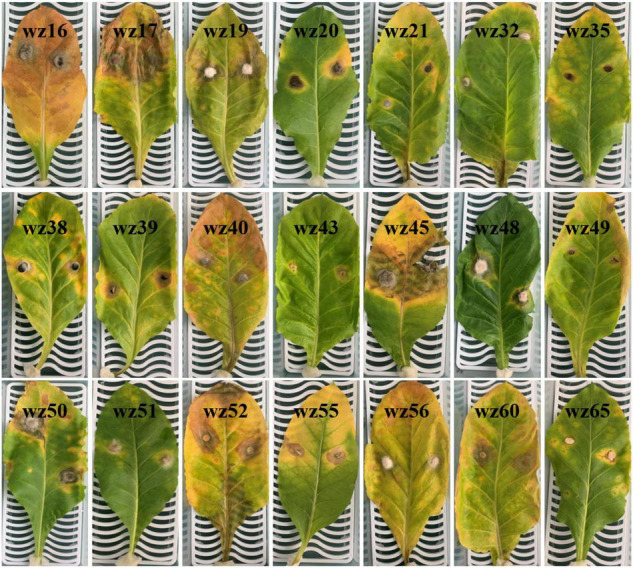
Symptoms on inoculated tobacco leaves at 10 days after inoculation and incubation at 28°C. These isolates came from symptomatic leaves and their identification based on ITS sequences is shown in Table 4. The white mesh board behind each leaf is the same size (25 cm × 10 cm).

A total of 47 fungal isolates belonging to 18 genera were obtained by tissue isolation, and their ITS sequences have been deposited in NCBI GenBank (Table 4). The pathogenicity of all 47 isolates was tested on detached tobacco leaves. Afterward, leaves were wounded, inoculated, and then incubated for 10 days at 28°C. The non-inoculated control and 26 isolates did not yield any symptoms on detached leaves, and the remaining 21 isolates caused varying degrees of symptoms on the detached leaves. Disease symptoms on detached leaves progressed from inoculation sites to surrounding areas and formed yellowish-brown spots with irregular shapes within a week (Figure 8). Among the 21 pathogenic isolates, six (wz16, wz17, wz19, wz45, wz50, and wz52) had strong pathogenicity (lesion area >3.14 cm^2^); seven (wz20, wz38, wz39, wz40, wz48, wz55, and wz60) had medium pathogenicity; and eight (wz21, wz32, wz35, wz43, wz49, wz51, wz56, and wz65) had weak pathogenicity (noticeable lesions <0.79 cm^2^). The above 21 strains were classified as *Colletotrichum* (wz35/49/51/52/55/60), *Xylariaceae* (wz21/38/40), *Corynespora* (wz16/17/45), *Pestalotiopsis* (wz19/56), *Alternaria* (wz20/39), *Epicoccum* (wz48/50), *Byssosphaeria* (wz43), *Phoma* (wz32), or *Diaporthe* (wz65). The symptoms caused by three of the six highly virulent isolates (wz16, wz17, and wz45) were consistent with those of originally collected diseased leaves, and these were all identified as *Corynespora cassiicola*.

**TABLE 4:** Fungi isolated from symptomatic leaves, and their identity based on ITS sequences, and their GenBank accession numbers.

Species	Strain name	Accession number	Species	Strain name	Accession number
*Alternaria* sp.	wz20	MW220839	*Epicoccum sorghinum*	wz48	MZ093627
*Alternaria* sp.	wz39	MZ093624	*E*. *sorghinum*	wz50	MW220861
*Annulohypoxylon* sp.	wz5	MW220860	*Marasmiellus* sp.	wz3	MW220847
*Arthrinium* sp.	wz33	MW220851	*Nigrospora* sp.	wz1	MW220834
*Byssosphaeria* sp.	wz43	MW220856	*Nigrospora* sp.	wz37	MW220854
*Cercophora* sp.	wz27	MW220845	*Nigrospora* sp.	wz54	MW220865
*Cercophora coronata*	wz53	MW220864	*Pestalotiopsis* sp.	wz10	MW220835
*Cladosporium* sp.	wz47	MW220859	*Pestalotiopsis* sp.	wz19	MW220838
*Colletotrichum* sp.	wz34	MW220852	*Pestalotiopsis* sp.	wz56	MW220867
*Colletotrichum* sp.	wz35	MW220853	*Phaeosphaeriopsis* sp.	wz18	MW220837
*Colletotrichum* sp.	wz49	MZ093628	*Phoma* sp.	wz22	MW220841
*Colletotrichum* sp.	wz60	MW220870	*Phoma* sp.	wz30	MW220848
*Colletotrichum* sp.	wz63	MW220872	*Phoma* sp.	wz32	MW220850
*Colletotrichum gloeosporioides*	wz51	MW220862	*Phoma* sp.	wz46	MW220858
*C*. *gloeosporioides*	wz52	MW220863	*Phytophthora nicotianae*	wz59	MW220869
*C*. *gloeosporioides*	wz55	MW220866	*Xylariaceae* sp.	wz21	MW220840
*C*. *gloeosporioides*	wz64	MW220873	*Xylariaceae* sp.	wz31	MW220849
*Corynespora cassiicola*	wz16	MZ093622	*Xylariaceae* sp.	wz38	MZ093623
*C*. *cassiicola*	wz17	MW220836	*Xylariaceae* sp.	wz40	MZ093625
*C*. *cassiicola*	wz45	MZ093626	*Xylariaceae* sp.	wz44	MW220857
*Diaporthe* sp.	wz4	MW220855	*Xylariaceae* sp.	wz57	MW220868
*Diaporthe* sp.	wz23	MW220842	*Xylariales* sp.	wz24	MW220843
*Diaporthe* sp.	wz25	MW220844	*Xylariales* sp.	wz28	MW220846
*Diaporthe* sp.	wz65	MW220874			

## Discussion

In the past, research on plant pathogens and phyllosphere microorganisms was often based on traditional culture-dependent methods, which may have underestimated the number of microbial communities and the functions of non-pathogenic and non-culturable microorganisms (Dissanayake et al., 2018). This study provided new insights into potential phyllosphere pathogens and into the microbial community structure and diversity in asymptomatic and symptomatic tobacco leaves. In the present study, we found a correlation between the health status of tobacco leaves and phyllosphere microbial community structure and composition, but this correlation was not very strong (*r* < 0.6). This finding implied that foliar disease may affect the phyllosphere by promoting or inhibiting the growth of certain microorganisms.

Among the dominant taxa, we identified a large number of bacterial and fungal genera which have been reported as phytopathogens or plant growth promoters, as well as a several neutral bacteria and fungi. The neutral bacterial genera such as *Cetobacterium*, *Providencia*, *Faecalibacterium*, and *Methylobacterium* have been reported from leaves of different plants as endophytes or epiphytes. In previous studies, *Sphingomonas*, *Bacillus*, and *Cyanobacteria* were considered to be beneficial to plant growth and to protect plants from pathogens (Innerebner et al., 2011; Han et al., 2016). But in our study, they were not detected from tobacco leaves. This difference may result from differences in climate and geography and in host species (Finkel et al., 2011).

*Pseudomonas*, *Pantoea*, and *Ralstonia* are typical phytopathogenic genera associated with tobacco. *Pseudomonas* as a pathogenic genus, has a wide host range (Morris et al., 2008). Wildfire disease of tobacco is caused by *Pseudomonas syringae* pv. *tabaci* (Ramegowda et al., 2013). Some *Pantoea* species can cause human diseases, while others can cause plant diseases (Cruz et al., 2007). Species of *Ralstonia* are pathogens causing bacterial wilt in many plants (Genin and Denny, 2012) including *R. solanacearum* causing tobacco bacterial wilt. PICRUSt predictions showed that the functions of most bacteria were associated with regular physiological activities, such as metabolism, genetic information processing, cellular processes, and organismal systems. Among bacterial communities, some species of *Pseudomonas*, *Pantoea*, and *Providencia* can cause human diseases, but it is uncommon for human pathogenic bacteria to be found on plant leaves, and we were only able to resolve to genera so the human pathogenic species might not have been present.

In contrast to bacteria, the fungal community contained more phytopathogenic genera. *Phoma* is an ubiquitous genus among plants, and 110 species are plant pathogens (Deb et al., 2020). They can invade many economically important crops, including wheat, tobacco, oilseed rape, and tea, among others. There were other fungi besides *Phoma*, including *Alternaria* species which are known to cause tobacco brown spot (Hou et al., 2016), *Cercospora nicotianae* which causes tobacco frogeye (Fajola and Alasoadura, 1973), and *Fusarium* species which cause tobacco wilt (LaMondia, 2015). In addition to these genera, *Cladosporium*, *Stagonosporopsis*, *Moesziomyces*, *Gibellulopsis*, *Phyllosticta*, *Epicoccum*, *Plectosphaerella*, and *Corynespora* have been reported to invade plants as phytopathogenic genera. Based on the functional predictions of FUNGuild, most fungal communities were plant pathogens and saprotrophs, and few communities were endophytes, parasites or animal pathogens. The structure of fungal communities on tobacco leaves also supported this conclusion.

Plants with a higher microbial diversity were less prone to pathogen invasion than those with less microbial diversity (Berg et al., 2017). This is likely due to more diverse microbial communities having higher competition for nutrient sources, free water and ecological niches than potential pathogens (Shade, 2017). Therefore, leaves with higher microbial diversity may be associated with reduce chances of disease outbreaks (Yang et al., 2017). Disease severity is one of the many factors that affect phyllosphere microorganisms. Tobacco leaves of different disease index infected by *Didymella segeticola* have different microbial diversity, and the relative abundance of dominant genera in the phyllosphere increased with increasing disease index (Huang et al., 2021). The abundance of Ascomycota and *Podosphaera* increased as pumpkin powdery mildew severity increased from L1 to L4, and the diversity and richness of the fungal community increased from L1 to L2, and then declined from L2 to L4 (Zhang et al., 2017). Therefore, if we had tested tobacco leaves with different levels of symptoms or different disease stages, their phyllosphere microbial community diversity may also have been different.

The traditional culture-dependent method yielded community composition results which differed from the metagenetic analysis. Among the 47 strains isolated from culturing, *Colletotrichum* was the dominant genus, which accounted for 19%, followed as *Xylariaceae* (13%), *Phoma* (9%), *Diaporthe* (9%), and *Cladosporium* (2%). We only checked for fungi growing up to 1 week, but a longer monitoring period such as up to 4 weeks may have revealed other fungal species. Based on the metagenetic analysis, the dominant fungi in the symptomatic leaves were found to be species of *Phoma* (35.05%), *Cladosporium* (23.87%) and *Alternaria* (6.88%), and the relative abundances of *Colletotrichum* and *Xylariaceae* were less than 0.01%. Therefore, the strains with high isolation frequency obtained by culture-dependent methods were not necessarily the dominant fungi in the leaf layer, and the strains with low isolation frequency may also be dominant fungi based on metagenetic analysis. Pathogenicity testing showed that in the same genus, some strains had strong pathogenicity, some strains had weak pathogenicity, and some strains even had no pathogenicity. In this study, we used wounded detached leaves for pathogenicity testing, which may have overestimated pathogenicity for opportunistic fungi, but in previous research in this lab, we have found that there were no significant differences in lesion size on attached leaves vs. wounded detached leaves in tests with a variety of tobacco pathogens (unpublished data). Some fungi isolated by culturing were not detected in metagenetic analysis, which may be because these fungi had low relative abundance or some other property such as prolific sporulation exaggerated their abundance. Therefore, the combination of metagenetic analysis and traditional culture-dependent methods perhaps can more comprehensively reflect the composition of phyllosphere microbial communities. Based on our previous observations of tobacco foliar diseases, we suspected that this was likely a fungal pathogen especially since the lesions were not vein limited, and hence we targeted fungi for isolation and did not attempt to culture bacteria. Future research will focus on confirming the causal agent or agents of this disease whether a bacterium may be involved.

## Conclusion

This study demonstrated that asymptomatic and symptomatic leaves of tobacco plants may differ in microbial community composition and potential pathogens. Based on isolation of culturable fungi from symptomatic leaves followed by pathogenicity testing, 21 potential pathogenic strains belonging to *Colletotrichum*, *Xylariaceae*, *Corynespora*, *Pestalotiopsis*, *Alternaria*, *Epicoccum*, *Byssosphaeria*, *Phoma*, and *Diaporthe* were found on symptomatic leaves, and the causal agent of this tobacco leaf spot may be *Corynespora cassiicola*. Metagenetic analysis of symptomatic leaves showed the following pathogen-containing genera on tobacco leaves: *Pseudomonas*, *Prevotella*, *Peptoniphilus*, *Gibellulopsis*, and *Meira*, among others. Metagenetic analysis also revealed that asymptomatic leaves harbored pathogenic genera such as *Pseudomonas*, *Pantoea*, *Cercospora*, and *Phaeosphaeriopsis* among others. Asymptomatic leaves had a higher microbial diversity and richness than symptomatic leaves. The combination of the traditional culture-dependent method and metagenetic analysis provided insights into the composition of microbial communities and potential pathogens on tobacco leaves more comprehensively than either one of these methods alone. These findings are significant for the prevention and control of tobacco diseases.

## Data Availability Statement

The datasets presented in this study can be found in online repositories. The names of the repository/repositories and accession number(s) can be found below: https://www.ncbi.nlm.nih.gov/genbank/, PRJNA722239.

## Author Contributions

L-GX, H-CW, and Z-HY contributed to conception, design of the study, and wrote the first draft of the manuscript. L-GX organized the database and performed the statistical analysis. TH wrote sections of the manuscript. All authors contributed to manuscript revision, read, and approved the submitted version.

## Conflict of Interest

The authors declare that the research was conducted in the absence of any commercial or financial relationships that could be construed as a potential conflict of interest.

## Publisher’s Note

All claims expressed in this article are solely those of the authors and do not necessarily represent those of their affiliated organizations, or those of the publisher, the editors and the reviewers. Any product that may be evaluated in this article, or claim that may be made by its manufacturer, is not guaranteed or endorsed by the publisher.

## Footnotes

1
https://github.com/UMNFuN/FUNGuild

2
https://github.com/picrust/picrust
